# Supplementary material for: Temperature Regimes Impact Coral Assemblages along Environmental Gradients on Lagoonal Reefs in Belize
Source: PLoS One. 2016 Sep 8;11(9):e0162098. doi: 10.1371/journal.pone.0162098 (PMC5015988; doi:10.1371/journal.pone.0162098)
Supplement: S1 Appendix — (DOCX) [file pone.0162098.s001.docx]

**S1 Appendix: Additional survey method details and AGRRA vs. video method comparison**

Six video transects (1 m x 20 m) were performed at each site using a GoPro® camera attached to a PVC stabilizing apparatus that allowed each diver to hold the camera steady with two hands while performing a transect. Six 20 m video transects per site has been shown to be sufficient to describe the coral community at a site ([Cruz *et al.*, 2008](#_ENREF_3)). Lead line of known length was attached to the camera rig to allow the diver to maintain a constant height above the substrate. Two lasers were placed on the camera rig 25 cm apart and were used to calibrate distances during video transect analysis. The entire apparatus, including the GoPro® camera, cost approximately $250, which is a more cost-effective option than commonly used stereo-video rigs that utilize much more expensive cameras and underwater housings. Video transects were analyzed in the same manner as the AGRRA transects. Length and width of each coral was recorded from measurements made while watching the video on a computer screen. The distance between the two lasers at each given stopped frame was used to calibrate the length and width measurements. Height of coral colonies was not recorded due to the two dimensional nature of the video recordings. Coral cover and coral density were also calculated using video transects. Video transects were calibrated in the field to be 1 m wide and 20 m long. However, due to shallow water and conditions at some sites the transects were less than 1 m wide, creating a slightly variable transect area, which was corrected for via the 25 cm laser scale.

All corals greater than 4 cm^2^ in area (as measured by a metric ruler) at least partially inside of the video screen were surveyed following AGRRA guidelines (AGRRA 2003). The genus and species of each coral was identified and number and size of individual colonies of each species were recorded on underwater data sheets. The outward facing surfaces of each colony were analyzed for health and mortality using parameters defined by AGRAA (live, pale, bleached, new mortality, old mortality). After the data were collected, species diversity, abundance, species richness, and coral life history ([Darling *et al.*, 2012](#_ENREF_4)) were calculated for each site.

The results of the two survey methods were analyzed separately and then compared. It was determined that the data from the two transect methods could be combined for species richness, abundance, and Shannon diversity, as survey method was not a significant factor in the ANOVA (Table 3A). Percent coral cover was calculated using video transect data only, as the AGRRA method over-estimates coral cover. This is due to the fact that the AGRRA methodology requires any coral that is even partially within the transect to be quantified in full, leading to over-estimates of coral cover. Coral density (# of corals/ m^2^) was also calculated from video transect data only.

Each method has downsides. AGRRA surveys are time consuming (1 hour per transect) and have the potential to overestimate coral cover. In addition, the diver must identify every coral individually while also maintaining buoyancy and safe diving practices, which can be difficult especially in rough weather. With video analysis, transects can be recorded much faster (less than 1 hour for 3 surveys) with approximately 150% of the AGRRA survey area covered using video in significantly less dive time. Videos were analyzed after the fact, allowing several researchers to analyze the video together and make a more thorough identification of coral species than underwater AGRRA surveys allow for. While limitations in video framing and the two dimensional nature of the video prevent accurate measurements of individual size, coral cover can also be estimated more accurately than with AGRRA methodology. Overall, video analysis requires more time than field transects, but has the potential to be more accurate as the time crunch or other external stressors that may be experienced underwater are no longer present. The two methods are comparable in terms of results, however video surveys are more efficient in the field and are have previously been shown to have the potential to be more accurate ([Lirman *et al.*, 2007](#_ENREF_6); [Turner *et al.*, 2015](#_ENREF_7)).

**References**

Cruz, I., Kikuchi, R.K. & Leão, Z.M. (2008) Use of the video transect method for characterizing the Itacolomis reefs, eastern Brazil. *Brazilian Journal of Oceanography*, **56**, 271-280.

Darling, E.S., Alvarez‐Filip, L., Oliver, T.A., McClanahan, T.R. & Côté, I.M. (2012) Evaluating life‐history strategies of reef corals from species traits. *Ecology Letters*, **15**, 1378-1386.

Lirman, D., Gracias, N., Gintert, B., Gleason, A., Reid, R., Negahdaripour, S. & Kramer, P. (2007) Development and application of a video-mosaic survey technology to document the status of coral reef communities. *Environmental monitoring and assessment*, **125**, 59-73.

Turner, J.A., Polunin, N.V., Field, S.N. & Wilson, S.K. (2015) Measuring coral size-frequency distribution using stereo video technology, a comparison with in situ measurements. *Environmental monitoring and assessment*, **187**, 1-10.
