# Supplementary figures and images for: Temperature Regimes Impact Coral Assemblages along Environmental Gradients on Lagoonal Reefs in Belize
Source: PLoS One. 2016 Sep 8;11(9):e0162098. doi: 10.1371/journal.pone.0162098 (PMC5015988; doi:10.1371/journal.pone.0162098)

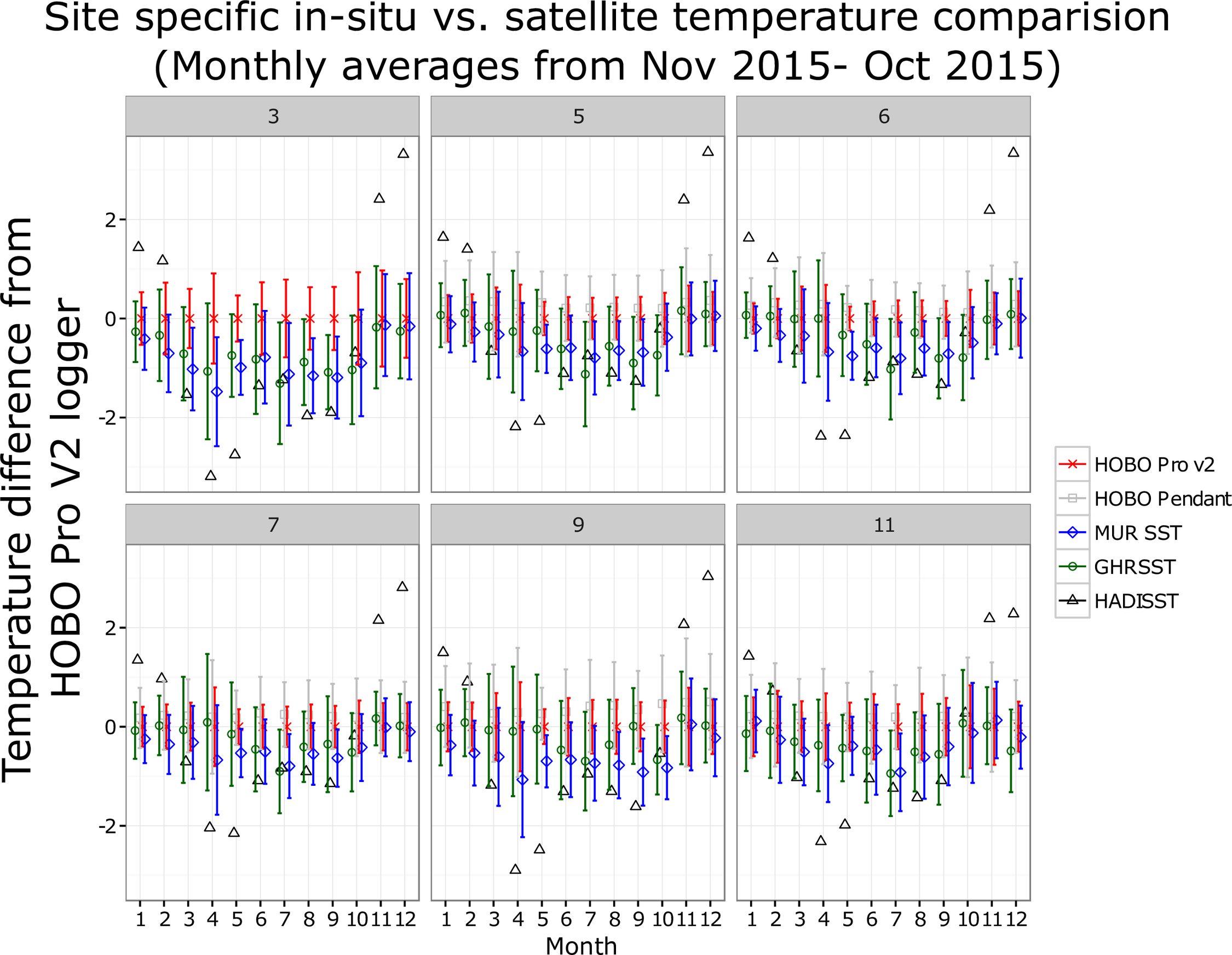

Supplement: S1 Fig — A comparison of in situ temperature and MUR SST. In situ loggers were collected from 6 sites along the BBRS (site numbers are listed in the gray headers above each panel). Each panel shows a month by month comparison of in situ logger measurements and SST products. Zero on the y-axis represents the average value for the Hobo Pro V2 loggers at each site. Red errors bars the standard deviation over a month for each logger. Gray squares show average values for an additional in situ logger that was placed at the site (± 1 standard deviation). Blue, green, and black symbols show monthly average values for various SST products (± 1 standard deviation). (TIF) [file pone.0162098.s002.tif]

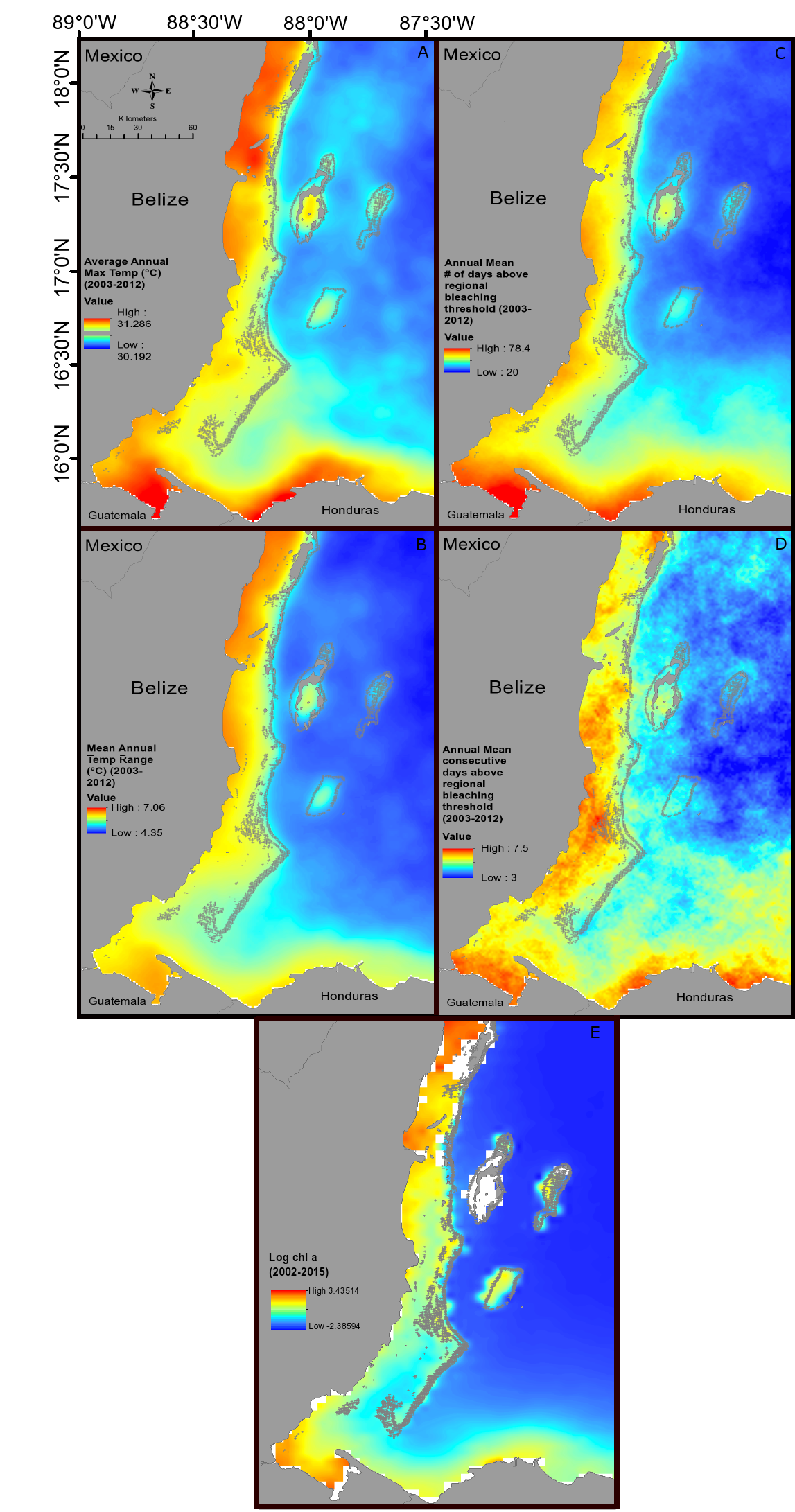

Supplement: S2 Fig — Maps showing the 4 parameters used to calculate site type: yearly maximum temperature (A), Mean annual temperature range (B), Annual mean number of days above the bleaching threshold (C),Annual mean consecutive days above the bleaching threshold (D), and 13 year mean chl-a concentration from 2002–2015 (E). Maps generated from means calculated from daily satellite measurements taken from Jan 2003-Dec 2012. (TIF) [file pone.0162098.s003.tif]

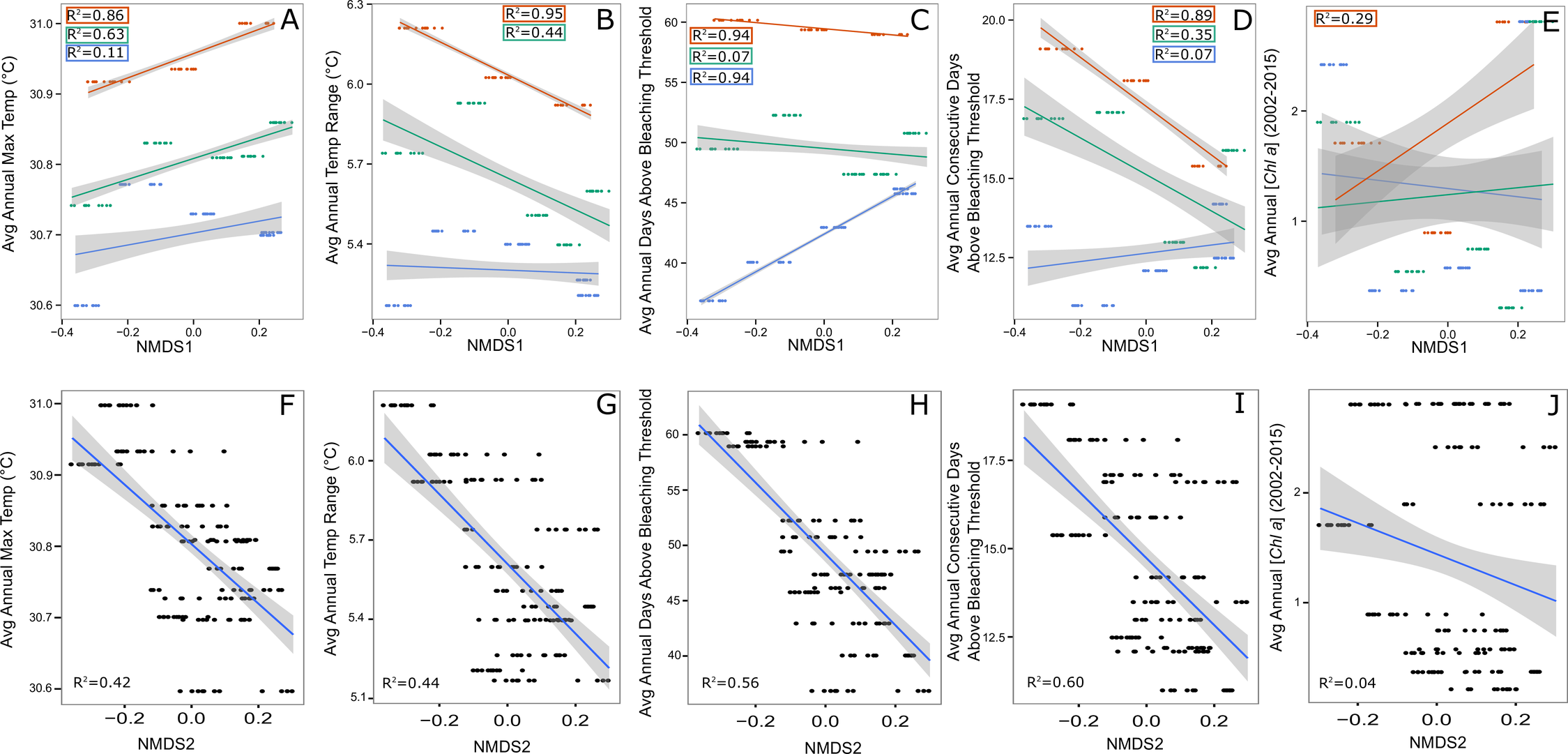

Supplement: S3 Fig — Linear regression of average annual max temp (A, F), average annual temp range (B, G), average annual days above the bleaching threshold (C, H), average annual consecutive days above the bleaching threshold (D, I), and Chl-a (E, J) vs. NMDS1 and NMDS2 by site type. R2 values are included for each regression that yielded a significant slope (p <0.05). (TIF) [file pone.0162098.s004.tif]
