# Supplementary material for: Temperature Regimes Impact Coral Assemblages along Environmental Gradients on Lagoonal Reefs in Belize
Source: PLoS One. 2016 Sep 8;11(9):e0162098. doi: 10.1371/journal.pone.0162098 (PMC5015988; doi:10.1371/journal.pone.0162098)
Supplement: S1 Table — Summary of survey sites, how they were classified, and where they were located (latitude/ longitude). (PDF) [file pone.0162098.s005.pdf]

S1 Table: Site locations

Site Name	Site Type	Latitude (Degrees N)	Longitude (Degrees W)	Depth	Habitat
1- Dangriga Low	Low_{TP}	17.078	88.01285	3-5 m	Back reef
2- Dangriga Mod	Mod_{TP}	16.99597	88.08416	3-5 m	Patch reef
3- Dangriga High	$High_{TP}$	16.79491	88.27699	3-5 m	Nearshore
4- Placencia Low	Low_{TP}	16.45816	88.01295	3-5 m	Back reef
5- Placencia Mod	Mod_{TP}	16.49995	88.16527	3-5 m	Patch reef
6- Placencia High	$High_{TP}$	16.4654	88.31315	3-5 m	Nearshore reef
7- Sapodilla Low	Low_{TP}	16.15729	88.25073	3-5 m	Back reef
8- Sapodilla Mod	Mod_{TP}	16.13013	88.33234	3-5 m	Patch reef
9- Sapodilla High	$High_{TP}$	16.2245	88.62943	3-5 m	Nearshore
10- Belize City Low	Low_{TP}	17.54239	88.06509	3-5 m	Back reef
11- Belize City Mod	Mod_{TP}	17.64363	88.0264	3-5 m	Patch reef
12- Caulker Low	Low_{TP}	17.79846	88.00196	3-5 m	Back reef
13- Caulker Mod	Mod_{TP}	17.82413	88.02581	3-5 m	Patch reef

S1 table: Summary of survey sites, site types, latitude, and longitude.
