# Supplementary material for: Temperature Regimes Impact Coral Assemblages along Environmental Gradients on Lagoonal Reefs in Belize
Source: PLoS One. 2016 Sep 8;11(9):e0162098. doi: 10.1371/journal.pone.0162098 (PMC5015988; doi:10.1371/journal.pone.0162098)
Supplement: S2 Table — Summary of p and R2 values for physical parameters vs. NMDS1 and NMDS 2. Significant p-values are in bold. (PDF) [file pone.0162098.s006.pdf]

S3 Table: *p*-values and R^2 from Linear Regression of Physical Parameters vs. NMDS1 and NMDS2

	NMDS1		NMDS2	
	p-value of slope	R^2	*p*-value of slope	R^2
Avg Annual Max Temp	**0.02**	0.0327	**<0.0001**	0.4154
Avg Annual Range	**0.0001**	0.0926	**<0.0001**	0.4361
Avg Annual Days Above Bleaching Threshold	**0.3**	0.0063	**<0.0001**	0.5644
Avg Annual Consecutive Days Above Bleaching Threshold	**<0.0001**	0.1026	**<0.0001**	0.6039
Chl a	0.5	0.0035	**0.009**	0.0434

S3 Table: Summary of *p*- and R^2 values for temperature and nutrient parameters vs. NMDS1 and NMDS 2. Significant *p*-values are in bold.
