# Supplementary material for: Temperature Regimes Impact Coral Assemblages along Environmental Gradients on Lagoonal Reefs in Belize
Source: PLoS One. 2016 Sep 8;11(9):e0162098. doi: 10.1371/journal.pone.0162098 (PMC5015988; doi:10.1371/journal.pone.0162098)
Supplement: S3 Table — Summary of p and R2 values for physical parameters vs. NMDS1 by site type. Significant p-values are in bold. (PDF) [file pone.0162098.s007.pdf]

S2 Table: *p*-values and R^2 for Linear Regression of Physical Parameters vs. NMDS1 by Site Type

	low_{TP}		mod_{TP}		$high_{TP}$	
	p-value of slope	R^2	*p*-value of slope	R^2	*p*-value of slope	R^2
Avg Annual Max Temp	**0.01**	0.1119	**<0.0001**	0.6268	**<0.0001**	0.8627
Avg Annual Range	0.442	0.0104	**<0.0001**	0.4389	**<0.0001**	0.9514
Avg Annual Days Above Bleaching Threshold	**<0.0001**	0.9514	**0.04**	0.9514	**<0.0001**	0.9435
Avg Annual Consecutive Days Above Bleaching Threshold	**0.05**	.06612	**<0.0001**	0.6664	**<0.0001**	0.8946
Chl a	0.6	0.0058	0.6	0.0046	**0.0006**	0.2878

Table S9: Summary of *p*- and R^2 values for physical parameters vs. NMDS1 by site type. Significant *p*-values are in bold.
