# Supplementary material for: Temperature Regimes Impact Coral Assemblages along Environmental Gradients on Lagoonal Reefs in Belize
Source: PLoS One. 2016 Sep 8;11(9):e0162098. doi: 10.1371/journal.pone.0162098 (PMC5015988; doi:10.1371/journal.pone.0162098)
Supplement: S4 Table — Populations of major towns in Belize from 2010–2015. Data source: Statistical Institute of Belize. (PDF) [file pone.0162098.s008.pdf]

City/Town	Census Population 2010	Estimated Mid-year Population 2010	Estimated Mid-year Population 2011	Estimated Mid-year Population 2012	Estimated Mid-year Population 2013	Estimated Mid-year Population 2014	Estimated Mid-year Population 2015
Orange Walk	13,708	13,707	13,704	13,700	13,696	13,692	13,687
San Pedro	11,767	11,884	12,749	13,637	14,549	15,484	16,444
Belize City	57,169	57,264	57,966	58,686	59,426	60,184	60,963
Belmopan	13,939	14,077	15,098	16,146	17,222	18,326	19,458
Dangriga	9,593	9,606	9,701	9,799	9,899	10,002	10,108
Punta Gorda	5,351	5,365	5,468	5,574	5,683	5,795	5,910
Country Total	322,453	323,598	332,084	340,792	349,728	358,899	368,310

Source: Statistical Institute of Belize (http://www.sib.org.bz/statistics/population)
